# NEIGHBORHOOD PROXIMITY AND SPATIAL SPILLOVER: IMPACT ON OLDER ADULT HEALTH

**DOI:** 10.1093/geroni/igae098.1502

**Published:** 2024-12-31

**Authors:** Michael Desjardins, Kyle Moored, Timothy Shields, Frank Curriero, Michelle Carlson

**Affiliations:** Johns Hopkins University, Baltimore, Maryland, United States; Johns Hopkins Bloomberg School of Public Health, Baltimore, Maryland, United States; Johns Hopkins University, Baltimore, Maryland, United States; Johns Hopkins University, Baltimore, Maryland, United States; Johns Hopkins University, Baltimore, Maryland, United States

## Abstract

Studying the impacts of neighborhoods on health exposures and outcomes has seen increased attention over the last few decades. Neighborhoods can describe where individuals work, live, and recreate in granular detail, facilitating our understanding of activity spaces throughout the lifecourse. However, a plethora of studies in neighborhood health, especially among older adults treat these spatial units as statistically independent. Most research in this domain fails to capture the potential influence of surrounding neighborhoods on health exposures and outcomes in the target neighborhood. Many of these studies also fail to check for residual spatial autocorrelation/dependence of modeling results, which may violate the assumption of independence that is fundamental in statistics. The explanatory power of regression models may also improve by accounting for the spatial dependence of the variables in question. We present conceptual examples (e.g., blue/green spaces, neighborhood socioeconomic status, and other social determinants of health) of spatial regression models that consider the influence of nearby neighborhoods on the dependent variable of interest. We also highlight the need to consider neighborhoods that are not adjacent since individuals may interact with neighborhoods farther away more often than those immediately adjacent. We then present case studies of modeling neighborhood and environmental predictors of cognitive outcomes of older adults in the Cardiovascular Health Study (CHS). Emphasis will also be on modeling lifecourse exposures. Our work highlights the power of spatial models to capture spillover and protective effects, which can greatly improve our understanding of older adult activity spaces and health across the lifecourse.

